# The Limited Impact of Low-Volume Recreational Dance on Three-Compartment Body Composition and Apparent Bone Mineral Density in Young Girls

**DOI:** 10.3390/children9030391

**Published:** 2022-03-10

**Authors:** Chiara Milanese, Valentina Cavedon, Ilaria Peluso, Elisabetta Toti, Carlo Zancanaro

**Affiliations:** 1 Laboratory of Anthropometry and Body Composition, Department of Neurosciences, Biomedicine and Movement Sciences, University of Verona, 37134 Verona, Italy; chiara.milanese@univr.it (C.M.); valentina.cavedon@univr.it (V.C.); 2 Research Centre for Food and Nutrition, Council for Agricultural Research and Economics (CREA-AN), 00178 Rome, Italy; ilaria.peluso@crea.gov.it (I.P.); elisabetta.toti@crea.gov.it (E.T.)

**Keywords:** physical exercise, body fat, body lean mass, bone mineral content, bone mineral density, dual-energy X-ray absorptiometry

## Abstract

Recreational dance is practiced worldwide as a multidimensional physical activity with a potential for prevention of a sedentary lifestyle and overweight/obesity. This study explored in young (7–15 year; *n* = 21) girls the effect of long-term (>1 year) exposure to recreational (2 h/w) dancing on three-compartment body composition. Recreational dancers (RD) were compared with recreational (≤4 h/w) artistic gymnasts (RG, *n* = 22) and physically active young girls not involved in structured extracurricular physical activity (control; C, *n* = 22), adjusting for confounding variables (age, body mass, menarche). We hypothesized for RD an intermediate body composition between RG and C. The three groups had similar age and body mass index. Body composition indices in RD were intermediate between that of C and RG, but RD values were not statistically significantly different vs. C. This agreed with the not statistically significant higher energy expenditure (MET-min/w) in RD vs. C (1357.7 ± 805.32 and 1090.9 ± 596.63, *p* = 0.172). In conclusion, long-term recreational dance exposure at low volume had limited positive effect on body composition of young girls vs. unstructured extracurricular physical activity. Future work will explore the potential of recreational dance at higher volume (3–4 h/w) to improve body composition in young girls.

## 1. Introduction

Physical activity positively affects body composition in children [1], and may be associated with better body composition later in life [2]. In turn, body composition is associated with physical fitness [3], i.e., the capacity to perform physical activity thereby implementing positive feedback. Moreover, the level of physical activity is positively associated with cognitive function and academic achievement in children [4], as well as attitudes (e.g., motivation, self-esteem [5]), and lowers the risk of developing cardiovascular diseases, mental disorders, and skeletal problems later in life [6]. Therefore, current international guidelines recommend that children limit sedentary behaviors and spend at least 60 min each day in moderate-to-vigorous-intensity physical activity [7]. Unfortunately, the majority of children fail to meet these guidelines [8]. A study carried out in eight European countries, inclusive of Italy, showed that only 9% of girls and 26% of boys performed ≥60 min/day of moderate-to-vigorous-intensity physical activity [9]. Since physical activity habits and attitudes are formed during childhood and adolescence and persist into adulthood [10], there is a need for effective interventions aimed at counteracting the ongoing reduction in habitual physical activity early in age [7], as well as increase physical activity and reduce sedentary behavior in children [11]. Reducing the prevalence of unhealthy body composition in children should be a target for effective interventions [12]. 

When analyzing components of body composition in children, fat mass (FM) and the associated fat-free mass are currently measured, especially concerning the amount of physical activity or its changes [13]. In the context of fat-free mass, bone mineral content (BMC) and the derived bone mineral density (BMD) have special relevance, because physical activity plays a crucial role among the environmental factors that affect bone mass acquisition in children [14]. It is generally assumed that mechanical loading associated with physical activity is an important determinant of skeletal growth and modeling [15], although the precise mode of action is not fully understood. Importantly, low BMD and osteoporosis in adulthood may have pediatric antecedents, as bone mass during growth is the foundation for the adult skeleton [16], and approximately 25% of the total adult mineral mass is gained during the peri-pubertal years [17]. However, the specific exercise regimen that elicits a biologically significant bone response at relevant sites remains unclear [18].

Recreational dance is a type of multidimensional physical activity which is practiced worldwide, especially by girls, and has the potential for the prevention of sedentary lifestyle and, therefore, overweight/obesity. Interestingly, dance programs demonstrated lower dropout rates than other fitness programs [19], possibly due to low cost and matching to multiple purposes (e.g., develop social skills, improve one’s physical fitness or motor coordination as well as mood, body image, well-being, and mental health [20]). Dancing participation can be taken up early in childhood, and it can provide entertainment all along life [21]. Accordingly, dance participation could be instrumental to limit the current trend to adverse changes in girls’ body composition, especially percent FM (%FM). Dance training is associated with positive cardiovascular and body composition adaptations in college-aged females even at moderate engagement [22]; further, recreational dance training involves some impact loading activity [22,23,24], thereby suggesting it may also have positive effects on mineral bone accrual. Increased BMD at the lumbar vertebrae and the hip was found in adolescent female dancers vs. non-exercising controls [25]. While the very large majority of girls practicing dance is recreational, the patterns of FM, fat-free mass, and bone mineral in children exposed to recreational dance has received little attention to date [26,27,28], at odds with young elite dancers [29,30,31,32,33,34,35]. Therefore, research is needed to clarify the body composition adaptations to recreational dance exposure in children.

Like dance, artistic gymnastics is largely participated by young girls and has a recognized influence on body FM as well as fat-free mass (inclusive of its bone mineral component [36,37]) showing differential effects vs. other sports [38]. Accordingly, artistic gymnastics appears a suitable benchmark when assessing the effects of recreational dance participation on body composition. Given that the focus of this investigation was on recreational dance, and considering that artistic gymnastics participation at the elite level may be associated with several issues also involving body composition [39], recreational gymnasts were only considered in the present study. The control group in this work comprised physically active girls not involved in structured extracurricular physical activity. In fact, given the low level of physical activity in the general pediatric population [8], using sedentary participants as the control may lead to misinterpretation of results [40] by artificially amplifying the effect of recreational physical activities. 

This study aimed at testing the hypothesis that long term (>1 year) exposure to low-volume recreational dance in the form of structured extracurricular physical activity is associated with intermediate body composition characteristics between recreational gymnasts (a benchmark of structured physical activity) and physically active controls not involved in structured extracurricular physical activity. That is, we sought for a possible differential effect of recreational dance vs. unstructured physical activity. 

Body composition was measured by dual-energy X-ray absorptiometry (DXA). DXA is radiological technique using X-ray beams at two different energies; it makes use of the differential attenuation of the X-ray beam at these two energies to calculate the bone mineral content and soft tissue composition in the scanned region [41]; it can measure FM (and its percentage), lean soft tissue mass [LSTM], and bone mineral in a single scan. DXA is recognized as a powerful, reliable tool to assess body composition at all ages. A disadvantage of DXA is the use of ionizing radiation; however, the effective dose incurred during DXA scanning is very small, and, consequently, DXA has gained a reputation as a simple and safe technique that can be used for children [42]. The standard DXA output includes estimations of FM (and its percentage), lean soft tissue mass (LSTM) as well as BMC and BMD. In this work, analysis of FM, LSTM, and bone mineral was carried out at the whole-body (WB) and regional level using total body DXA scans. The bone mineral was also investigated at specific sites, i.e., the lumbar vertebrae and the hip as these are relevant targets for physical exercise [43]. Participants were recruited across a relatively large age range (7–15 year) to follow development spanning pre-pubertal and pubertal growth. 

## 2. Materials and Methods

### 2.1. Participants

#### 2.1.1. Recruitment

A convenience sample of Caucasian dancers and gymnasts was recruited by advertising at local gymnastics and dance facilities and by word-of-mouth. The study received approval from the ethics committee at the Department of Neurosciences Biomedicine and Movement Sciences (Prot. N. 33726/2013). Written, informed consent was obtained from girls and their parents. Inclusion criteria were: 7 year < age <16 year; stature, body mass, and body mass index (BMI) within the 3rd and 97th percentile of for the reference population in northern-central Italy [44]; regular participation (at least 2 h/w) for at least 1 year in dancing (both classic and modern) or regular participation (up to 4 h/w) for at least 1 year in artistic gymnastics. Participants in the control group had to be involved in habitual extracurricular physical activity for at least 2 h/w (excluding high impact loading sports) and energy expenditure of at least 600 metabolic equivalent of task-minute per week (MET-m/w) (vide infra). Exclusion criteria were: the presence of concurrent musculoskeletal pathology; recent (within the previous 6 months) injury; any ongoing pharmacological treatment. Girls with oligo/amenorrhea (no menses for ≥3 months) were excluded since they are at risk of not accruing bone at the expected rate [45]. In summary, three groups entered the study: recreational dancers (RD), recreational gymnasts (RG), and physically active controls (C).

#### 2.1.2. Collection of Participants’ Characteristics

Menarche and menstrual status were determined by questionnaire [46]. Energy expenditure was indirectly estimated by physical activity records [47] and expressed as MET-min/w. MET is a practical procedure for expressing the energy cost of physical activities as a multiple of the resting metabolic rate [48]. All participants were asked to record as precisely and conscientiously as possible all physical activity for 7 days in a record designed for this study. The expert interviewer assigned each activity a five-digit code from the Compendium of Physical Activity that reflects the type and MET level intensity of activity performed [49]. A 24 h dietary recall was administered to assess caloric intake and its composition in terms of protein, lipid, and carbohydrate [50]. Habitual diet was assessed by a single-day dietary record as fully described by Willett [51]. Subjects were asked to report and describe in detail all food, beverages, and supplements consumed and to record the amounts of food consumed by food weighting or with the help of food labels to increase the accuracy of portion size. To ensure accuracy, subjects were encouraged to maintain their normal eating habits during the day of collection of the meals. The nutritional composition of the diet was calculated using the CREA-AN tables of the composition of foods [52] (https://www.crea.gov.it/-/tabella-di-composizione-degli-alimenti) (accessed on 15 September 2021). 

An estimation of the impact loading to which dancers and gymnasts were exposed was obtained by registering the number of maneuvers in a typical training session. This was accomplished by recording all training sessions in a typical week using two digital cameras (Panasonic Lumix DMC-TZ8) placed on a tripod and registering off-line each maneuver according to a dual criterion: low-impact loading or high impact loading. Maneuvers were registered as low impact loading if they consisted of movements where one foot is off the ground at a time or, one or both arms come in contact with the ground/exerciser without previous flight phase. High impact loading maneuvers consisted of movements where both feet are off the ground at the same time, or where one or both arms come in contact with the ground/exerciser following a previous flight phase. For dancers, low impact loading exercise typically involved movements such as battement tendu, rond de jambe, par terre and pirouettes; impact loading exercise typically involved jumping (temps sauté, pas échappé, pas assemble, glissade, grand jeté, sissonne, cabriole). For dancers in the age range 7–11 year, the average repetitions were 450/w and 200/w for low impact loading and impact loading movements, respectively. In the age range 12–15 year these figures were 800/w and 230/w, respectively. For recreational gymnasts, low impact loading exercise typically involved movements such as split, cast, turn on one foot; impact loading exercise typically involved movements such as forward roll, handstand, back handspring, split leap, front and back walkover, somersault, cartwheel. The average repetitions were 562/w and 964/w for low- and high-impact loading movements, respectively. 

### 2.2. Anthropometry and Body Composition 

Body mass was taken at the nearest 0.1 kg with an electronic scale (Tanita electronic scale BWB-800 MA, Wunder SA.BI. Srl, Milano, Italy ); stature was measured with a Harpenden stadiometer (Holtain Ltd., Crymych, Pembs, UK) at the nearest mm according to standard procedures [53]; BMI was calculated as body mass (kg)/height (m^2^).

Body composition was evaluated using a total body DXA scanner (QDR Explorer W, Hologic, MA, USA; fan-bean technology, software for Windows XP version 12.6.1). Quality control was carried out daily against a reference phantom supplied by the manufacturer to avoid possible baseline drift. All analyses were performed by the same operator to ensure consistency. Scans were performed in the late morning in a post-absorptive state. Participants were asked to refrain from vigorous exercise for at least 24 h before they arrived at the laboratory. Participants wore light-weight clothing with no metal or reflective material and removed all metal accessories. All measurements were performed in a supine position according to the manufacturer’s protocol. Scans were taken at WB, left hip, and lumbar spine (L1–L4) in the posteroanterior projection. During the WB scan, Velcro restraints were applied around participants’ ankles to ensure there was no movement during the scan. The radiation dose for the WB, lumbar spine and hip scans were 15.3, 2.2, and 2.2 cGy*cm^2^, respectively. According to the convention of the International Society for Clinical Densitometry [54], in vivo short-term precision for the DXA scans was calculated with repositioning and was 2.3%, 2.8%, 0.5%, 1.14 and 0.9%, for FM, %FM, LSTM, BMC, and BMD, respectively. For measurements at the lumbar spine (L1–L4) and total hip, precision was 1.43% and 1.28%, and 1.27% and 1.28%, for BMC and BMD, respectively. In WB scans, Hologic software readings divided the body into the trunk, entire arm (left and right), entire leg (left and right), and head. According to current recommendations [42,55], the BMC and BMD values considered for analysis were those in the total body less head region (TBLH) because the skull contains a large amount of total body mineral and is insensitive to physical activity [56]. In addition to the standard DXA output, the Appendicular (sum of arms and legs) FM and LSTM were also calculated. The Arms (average of right and left arm), Legs (average of right and left leg), and Appendicular (average of right arm and leg, and left arm and leg) BMC were calculated as well. 

### 2.3. Body Composition Indices and Bone Mineral Apparent Density

In this work carried out in growing girls, age, stature, and body mass were obvious confounding variables, especially for bone measurements, because DXA is a projectional technique and DXA-derived measurements do not adequately correct for body and/or bone size [57,58]. Accordingly, soft tissue body composition variables were expressed as a proportion to total body mass (%FM) or normalized by square stature (fat mass index, FMI; fat-free mass index, FFMI) [59,60]. The bone mineral was expressed as bone mineral apparent density (BMAD, g/cm^3^) according to the formula proposed by Katzman et al. [61]: BMAD = BMC/(bone area^2^/body stature). For lumbar spine BMAD calculation, the formula was: BMAD = BMC/(bone area)^1.5^ [62]. Z-scores were interpreted according to Crabtree and colleagues [55]. 

### 2.4. Statistical Analysis

A priori analysis of sample size was carried out using G*Power [v 3.1; [63]]. With a large effect size of 0.4 [64], a power of 0.80, three groups, and three covariates, the total sample size required for analysis of covariance (ANCOVA) was n = 64. Descriptive statistics are reported as means ± SD. Demographic characteristics, MET-min/w, and Z-scores were compared in the three groups with one-way analysis of variance (ANOVA) followed by post hoc analysis where needed. The Levene’s test was used to assess the equality of error variance. In the case of non-significance, post hoc comparisons were carried out with Bonferroni correction; in the case of significance, the Games–Howell test was used. Comparison of variables in the three groups of young girls was carried out in the general linear model (GLM) using the univariate ANOVA option. The main effect of Group (C, RD, RG) factor was assessed while adjusting for confounding variables, i.e., age (in months), body mass, and menarche (yes/no). The effect size was evaluated according to Cohen [64] using eta squared (ƞ^2^): small, 0.01; medium, 0.06; large, 0.14. Post hoc comparisons were carried out on estimated marginal means with Bonferroni correction. The normality of residuals was checked by visual inspection, Q-Q graphs, and the Kolmogorov–Smirnov test. Partial correlation (PC) analysis was carried out with Pearson’s r (r_PC_). The statistical package IBM-SPSS v.25 was used for all analyses. Statistical significance was set at *p* ≤ 0.05. 

## 3. Results

### 3.1. Characteristics of the Participants

A total of 73 participants were recruited. Eight participants did not complete all measurements and dropped out of the study. Accordingly, analysis was carried out of 65 participants (age, 134.8 ± 25.97 months [range, 93–202]; body mass, 38.8 ± 11.98 kg [range, 19.7–65.0]; stature, 144.6 ± 13.44 cm [range, 121.0–172.1]; BMI, 18.1 ± 2.86 kg/m^2^ [range, 13.4–25.0]) and the study resulted sufficiently powered. Participants were distributed in three groups of similar numerosity: C, *n* = 22; RD, *n* = 21; RG, *n* = 22. In RD, dance experience was 5.0 ± 2.61 year. In RG, gymnastics experience was 3.3 ± 2.13 year.

### 3.2. Characteristics of the Three Groups (C, RD, RG)

The three groups were not statistically different for age and BMI. The three groups were statistically significantly different for body mass and stature (Table 1). Post hoc analysis showed statistically significant higher body mass in C vs. RG (*p* = 0.036). Stature was statistically significantly higher in both C and RD vs. RG (*p* = 0.003 and *p* = 0.026, respectively).

**Figure 1:**
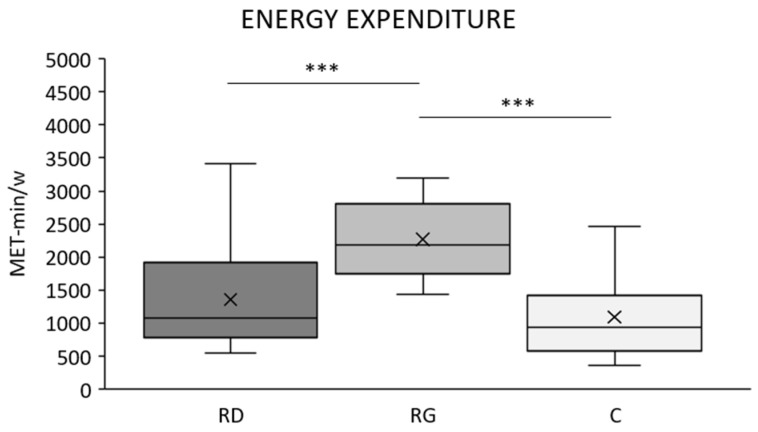
Box plots of estimated weekly energy expenditure (MET-min/w) in the three groups of young girls. RD, recreational dancers; RG, recreational gymnasts; C, physically active controls. ***, *p* ≤ 0.001. Box, range between the 25th and 75th percentile; solid line, median value; x, mean value; upper whisker, maximum score; lower whisker, minimum score.

The three groups were statistically significant different for energy expenditure (MET-min/w): F_(2,62)_ = 34.640, *p* < 0.001). Post hoc analysis (Figure 1) showed that energy expenditure in RG was statistically significant higher vs. C and RD (*p* < 0.001 for both), but not in RD vs. C (*p* = 0.172). Results of one-day dietary recall showed that diet composition was not statically significant different in the three groups: protein: C, 16.7 ± 5.12% g; RD, 16.4 ± 4.06%; RG, 19.2 ± 4.19%; F_(2,62)_ = 0.880, *p* = 0.423; lipid: C, 31.5 ± 5.91%; RD, 30.0 ± 6.05%; RG, 27.0 ± 3.34%; F_(2,62)_ = 1.434, *p* = 0.250; carbohydrate: C, 51.7 ± 8.45%; RD, 53,6 ± 7.78; RG, 53.7 ± 4.68%; F_(2,62)_ = 0.320, *p* = 0.728. Z-scores for WB, hip, and spine showed mineralization within the expected range for age (Z-score > −2) for all participants and were not statistically significant different in the three groups (Table 2).

Age at menarche was similar in the three groups: C, 11.6 ± 0.48 year; RD, 11.6 ± 0.62 year; RG, 11.6 ± 0.17 year, F_(2,21)_ = 0.128, *p* = 0.880. 

The descriptive body composition characteristics of the three groups of girls are presented in Supplementary Material Table S1.

#### 3.2.1. Comparison of Body Composition Indices in the Three Groups of Girls

One-way ANCOVA was run in the General Linear Model for all relevant body composition variables with group (C, RD, RG) as the factor and age (months), body mass (scale), and menarche as covariates.

#### 3.2.2. Fat Mass Percentage and Fat Mass Indices

**Figure 2:**
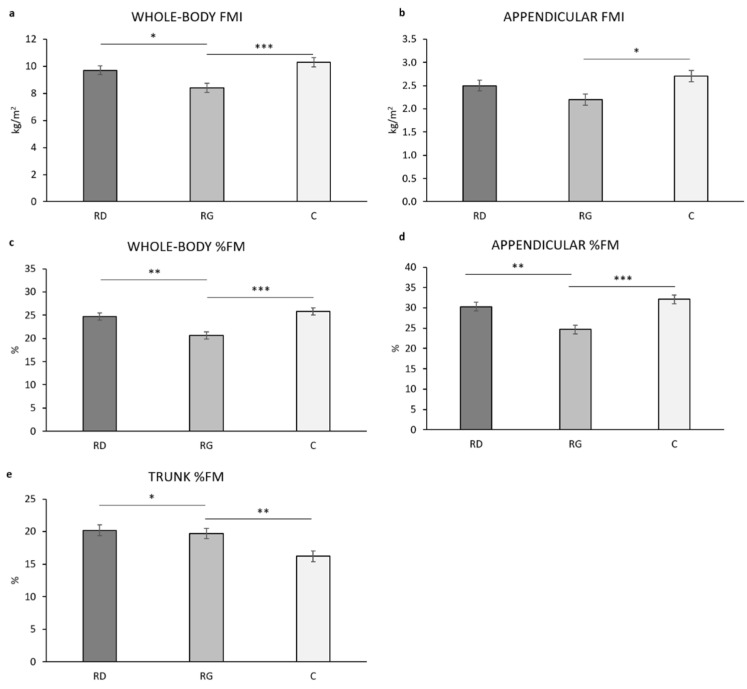
Estimated marginal means for fat mass index (FMI, kg/squared stature in meters; panel **a**, **b**) and percentage of fat mass (%FM; panel **c**–**e**) in the three groups of girls after adjusting for age, body mass, and menarche. RD, recreational dancers; RG, recreational gymnasts; C, physically active controls. *, *p* ≤ 0.05; **, *p* ≤ 0.01; ***, *p* ≤ 0.001.

A statistically significant main effect of group was present for the WB FMI (F_(2,59)_ = 7.896, *p* = 0.001, ƞ^2^ = 0.211). A post hoc analysis showed that the WB FMI is statistically significantly lower in RG vs. both C (*p* = 0.001) and RD (*p* = 0.019) (Figure 2a). The Appendicular FMI was statistically significant different in the three groups (F_(2,59)_ = 4.734, *p* = 0.012, ƞ^2^ = 0.138). A post hoc analysis showed that the Appendicular FMI is statistically significantly lower in RG vs. C (*p* = 0.013) but not RD (*p* = 0.090) (Figure 2b). The %FM was statistically significant different in the three groups at the WB, Appendicular, and trunk (F_(2,59)_ = 6.846, *p* = 0.002, ƞ^2^ = 0.188; F_(2,59)_ 11.115, *p* < 0.001, ƞ^2^ = 0.274; F_(2,59)_ 12.131, *p* < 0.001, ƞ^2^ = 0.291, respectively). A post hoc analysis showed that the WB FM, Appendicular FM, and trunk %FM are statistically significant lower in RG vs. both C and RD (*p* < 0.001, *p* = 0.002; *p* < 0.001, *p* = 0.002; *p* = 0.004, *p* = 0.012, respectively) (Figure 2c–e). Age and body mass showed a statistically significant main effect in all models (0.001 < *p* <0.022). Menarche showed a limited main effect for WB FM (*p* = 0.026).

#### 3.2.3. Lean Soft Tissue Mass Indices

**Figure 3:**
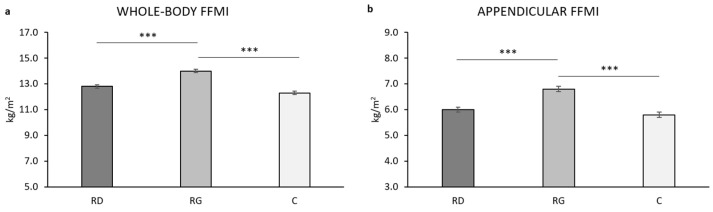
Estimated marginal means for fat-free mass index (FFMI, kg/squared stature in meters) at whole body (panel **a**) and appendicular (panel **b**) level in the three groups of girls after adjusting for age, body mass, and menarche. RD, recreational dancers; RG, recreational gymnasts; C, physically active controls. ***, *p* ≤ 0.001.

The WB FFMI was statistically significant different in the three groups of young girls (F_(2,59)_ = 36.572, *p* < 0.001, ƞ^2^ = 0.554). A post hoc analysis showed that WB FFMI is statistically significant higher in RG vs. both C (*p* < 0.001) and RD (*p* < 0.001) (Figure 3a). The Appendicular FFMI was statistically significant different in the three groups of young girls (F_(2,59)_ = 26.681, *p* < 0.001, ƞ^2^ = 0.475). The post hoc analysis showed that the Appendicular FFMI is statistically significant higher in RG vs. both C (*p* < 0.001) and RD (*p* < 0.001) (Figure 3b). Age showed a statistically significant main effect for the WB FFMI (*p* = 0.050) but not the Appendicular FFMI (*p* = 0.786). Body mass showed a statistically significant main effect for either WB or Appendicular FFMI (*p* < 0.001 for both). Menarche had no effect (*p* > 0.125 for both).

#### 3.2.4. BMAD

**Figure 4:**
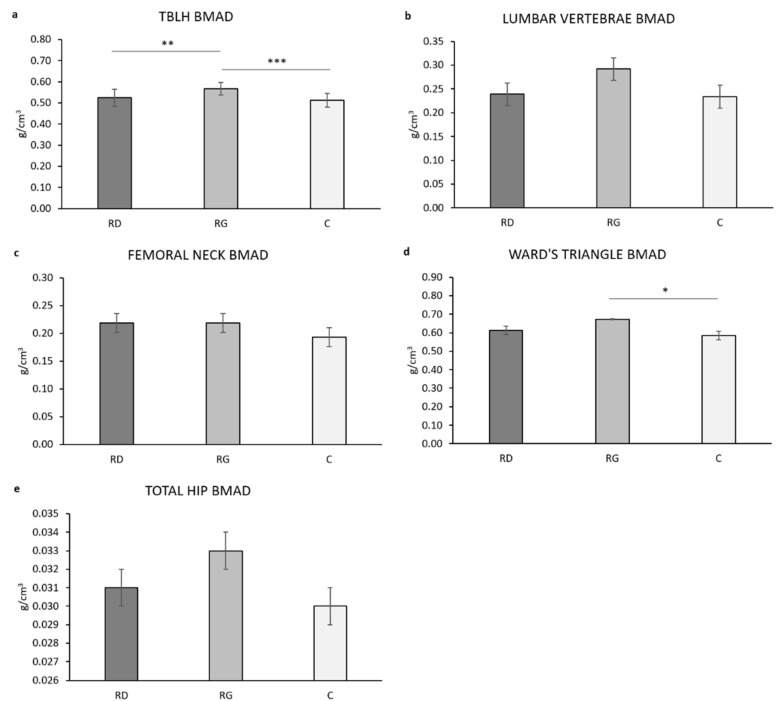
Estimated marginal means for bone mineral apparent density (BMAD, kg/cm^3^) in the three groups of girls at several skeletal sites (panel **a**–**e**) after adjusting for age, body mass, and menarche. RD, recreational dancers; RG, recreational gymnasts; C, physically active controls. *, *p* ≤ 0.05; **, *p* ≤ 0.01; ***, *p* ≤ 0.001. TBLH, total body less head.

The TBLH BMAD was statistically significant different in the three groups (F_(2,59)_ = 9.864, *p* < 0.001, ƞ^2^ = 0.251). A post hoc analysis showed that RG have a significantly higher TBLH BMAD vs. both C (*p* < 0.001) and RD (*p* = 0.004) (Figure 4a). Arms BMAD was statistically significant different in the three groups (F_(2,59)_ = 4.211, *p* = 0.020, ƞ^2^ = 0.125). A post hoc analysis showed that RG have significantly higher Arms BMAD vs. C (*p* = 0.018) but not RD (*p* = 0.181). Legs BMAD was statistically significant different in the three groups (F_(2,59)_ = 7.296, *p* = 0.001, ƞ^2^ = 0.198l). 

A post hoc analysis showed that RG has significantly higher Legs BMAD vs. both C (*p* = 0.001) and RD (*p* = 0.044). Appendicular BMAD was statistically significant different in the three groups (F_(2,59)_ = 5.401, *p* = 0.007, ƞ^2^ = 0.155). Post hoc analysis showed that RG has significantly higher Appendicular BMAD vs. C (*p* = 0.006) but not RD (*p* = 0.125). Age, body mass, and menarche showed no main effect on the dependent variable in any model (*p* > 0.183). 

Lumbar vertebrae BMAD (Figure 4b) was not statistically significant different in the three groups (F_(2,59)_ = 1.655, *p* = 0.200, ƞ^2^ = 0.053) with age showing a significant effect (*p* = 0.029) and body mass and menarche showing no effect (*p* > 0.220 for both). Femoral neck BMAD (Figure 4c) was not statistically significant different in the three groups (F_(2,59)_ = 0.750, *p* = 0.477, ƞ^2^ = 0.025), with age, body mass, and menarche showing no effect (*p* > 0.145 for all). Troch BMAD was not statistically significant different in the three groups (F_(2,59)_ = 0.527, *p* = 0.593, ƞ^2^ = 0.0.018) with age (F = 10.307, *p* = 0.002, ƞ^2^ = 0.149); body mass, and menarche showing no effect (*p* > 0.290 for both). Intertrochanteric BMAD was not statistically significant different in the three groups (F_(2,59)_ = 3.085, *p* = 0.053, ƞ^2^ = 0.095) with age, body mass, and menarche showing no effect (*p* > 0.204 for all). Ward’s BMAD was statistically significant different in the three groups (F_(2,59)_ = 3.751, *p* = 0.029, ƞ^2^ = 0.113) with age, body mass (F = 5.209 *p* = 0.026, ƞ^2^ = 0.081), and menarche showing no effect (*p* > 0.424 for all). A post hoc analysis showed that RG has significantly higher Ward’s BMAD vs. C (*p* = 0.028) but not vs. RD (*p* = 0.210) (Figure 4d). Total hip BMAD (Figure 4e) was not statistically significant different in the three groups (F_(2,59)_ = 2.063, *p* = 0.136, ƞ^2^ = 0.065) with age (F = 4.143, *p* = 0.046, ƞ^2^ = 0.066), body mass, and menarche showing no effect (*p* > 0.496 for all). 

#### 3.2.5. Correlation Analysis

**Figure 5:**
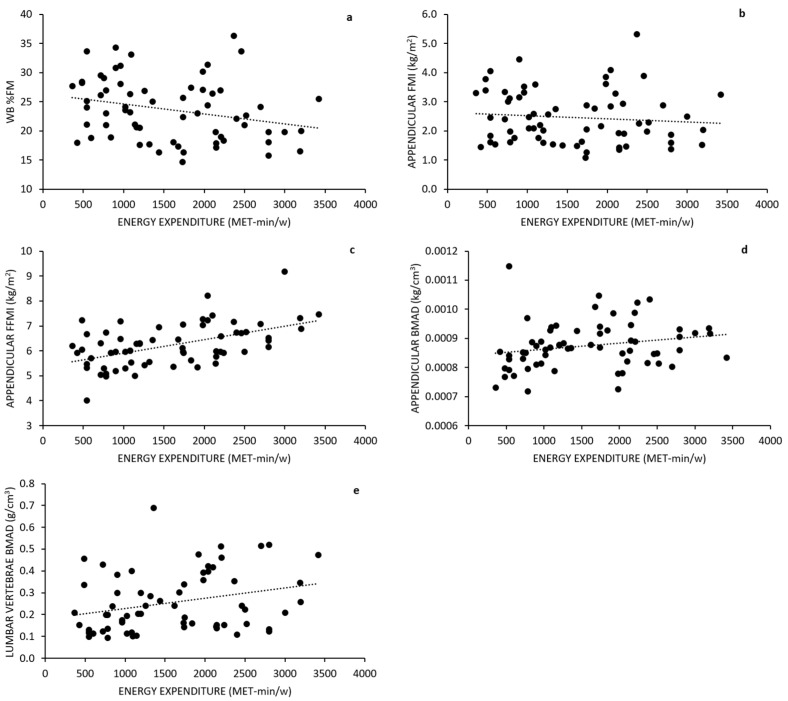
Scatterplot of weekly energy expenditure and percent FM, and stature-adjusted body composition variables in the whole groups of girls (recreational dancers, recreational gymnasts, and physically active controls). Raw correlations were as follows: (**a**) r = −0.262, *p* = 0.035; (**b**) r = −0.097, *p* = 0.443; (**c**) r = 0.516, *p* < 0.001; (**d**) r = 0.214; *p* = 0.087; (**e**) r = 0.353, *p* = 0.004. For adjusted correlations (r_PC_), see text. TBLH, total body less head. WB, whole body; FM, fat mass; FMI, fat mass index; FFMI, fat-free mass index; BMAD, bone mineral apparent density.

A partial correlation analysis showed that, after adjusting for age, stature and body mass, MET-min/w statistically significantly correlated with WB %FM (r = −0.556, *p* < 0.001) and Appendicular %FM (r = −0.554, *p* < 0.001). After adjusting for age and body mass, MET-min/w statistically significant correlated with WB FMI (r_PC_ = −0.393, *p* = 0.001), Appendicular FMI (r_PC_ = −0.364, *p* = 0.003), trunk %FM (r_PC_ = −0.366, P = 0.003; WB FFMI (r_PC_ = 0.557, *p* < 0.001), Appendicular FFMI (r_PC_ = 0.586, *p* < 0.001), TBLH BMAD (r_PC_ = 0.388, *p* = 0.002), Appendicular BMAD (r = 0.276, *p* =0.028)**,** Ward’s area BMAD (r_PC_ = 0.301, *p* = 0.016 and, at the limit of statistical significance, total lumbar vertebrae BMAD (r_PC_ = 0.245, *p* = 0.053) The scatterplots for the unadjusted relationship between MET-min/w and several body composition indices as well as BMAD variables are presented in Figure 5a–e.

## 4. Discussion

This work explored in girls aged 7–15 year the effect of long-term moderate (2 h/w) exposure to recreational dance participation on DXA-measured body composition in comparison with recreational gymnasts and physically active controls of similar age and BMI, controlling for several confounding variables. According to the IPAQ guidelines [65], C and RD were in the “moderate” category of energy expenditure (>600 <1200 MET-min/w, RG in the “high” category (>1200 MET-min/w). Therefore, the C group should properly be considered physically active.

Data presented herein show that the average body composition of the three groups of girls was in line with the Caucasian population [66,67]. In particular, average WB %FM for dancers (25.3) and gymnast (17.4) was similar (or lower) to previously reported figures in comparable populations [68,69] and was compatible with the girls’ recreational level of physical activity participation. Therefore, we are made confident that our sample is well suited to highlight the differential effect of the type of physical activity practiced by the three groups of girls.

The results of this work showed that, after adjusting for stature, age, body mass, and menarche, the average body composition indices of recreational dancers are typically intermediate between that of physically active controls and recreational gymnasts. However, mean values of body composition indices were, in general, statistically significantly different vs. recreational gymnasts, but not physically active controls, thereby not fully confirming our initial hypothesis of an intermediate body composition for recreational dancers.

All stature-independent indices of adiposity and muscularity (%FM, FMI, and FFMI) at the WB and regional level (Figure 2, Figure 3 and Figure 4) were statistically significantly different in the three groups of young girls after accounting for age, body mass, and menarche, with RG generally showing lower adiposity and higher muscularity vs. both C and RD. This was consistent with the higher absolute amount of physical activity (MET-min/w) in RG vs. both C and RD (*p* < 0.001 for both; Figure 1) and, in the whole sample of girls (*n* = 65), the negative relationship of MET-min/w with %FM after adjusting for age, body mass and stature (r_PC_ = −0.556, *p* < 0.001) as well as the positive correlation with appendicular FFMI after adjusting for age and body mass (r_PC_ = 0.586, *p* < 0.001). All average values of body composition indices in RD were intermediate between those of RG and C, suggesting a potential for recreational dance to induce better body composition vs. physically active control girls. However, such an effect was not statistically significant (post hoc *p* value > 0.05 for all indices). This is in accordance with RD showing a not statistically significant (*p* = 0.172) higher energy expenditure vs. C (about +300 MET-min/w). Such a limited difference in energy expenditure was probably unable to induce significant body composition improvement in RD vs. C. For FMI, all ANCOVAs showed a main, statistically significant, positive effect of both age and body mass, indicating a stature-independent role of these two variables in FM accrual from 8 to 15 year. For FFMI, a main, statistically significant, positive effect of body mass was found on both WB- and Appendicular FFMI; age showed a similar effect on WB, but not Appendicular FFMI, a proxy of body skeletal muscle [70]; this suggests that stature-adjusted skeletal muscle mass is invariant to age from 8 to 15 year in accordance with [67]. Menarche showed no statistically significant main effect on %FM, FMI, and FFMI indicating a limited impact for sexual maturation on such indices. 

TBLH, Arms, Legs, and Appendicular BMAD were statistically significantly different in the three groups of young girls after accounting for age, body mass, and menarche, with RG generally showing higher bone density vs. both C and RD. This is in agreement with the much higher (about fourfold) number of weekly high impact loading movements in RG vs. RD (see Section 2.1.2) and the absence of high impact loading movements in C. RD had higher average BMAD values than C, but the difference was not statistically significant (*p* > 0.05 for all). Interestingly, the difference between Legs BMAD in RG and RD was at the limit of statistically significance (*p* = 0.044). Since recreational dance especially imply impact loading on lower limbs [22,23,24], this suggests a potential for recreational dance to improve strength in leg bones. All ANCOVAs showed no main effect of age, body mass, and menarche, indicating that stature-adjusted bone density at these sites is independent of chronological age, body mass loading, and sexual maturation in our sample of young girls aged 7–15 year. 

Lumbar vertebrae (L1–L4) BMAD was statistically significant different in the three groups; however, post hoc analysis showed no significant between-group difference, despite a clearly higher average value in RG. RD and C showed closely similar values, suggesting that recreational dance had no major effect on bone density at the lumbar vertebrae level. However, in the whole sample of girls (*n* = 65) a positive age- and body mass adjusted correlation was found between MET-min/w and TBLH BMAD (r_PC_ = 0.388, *p* = 0.002), Appendicular BMAD (r_PC_ = 0.276, *p* = 0.028), and total lumbar vertebrae BMAD (r_PC_ = 0.288, *p* 0.023), confirming the positive, dose dependent effect of physical activity on bone strength during growth [71,72]. At the hip, BMAD was similar in the three groups at all specific sites (trochanter, intertrochanter, femoral neck) as well as the total hip, apart from the Ward’s triangle where BMAD was statistically significant higher in RG vs. C (*p* = 0.028) but not RD (*p* = 0.210). Interestingly, adjusted BMAD average value at the femoral neck was identical in RD and RG (0.219 g/cm^3^), a 13.5% higher value than C, indicating a possible positive effect of recreational dance at this site as found with moderate intensity, school-based physical activity intervention [73]. All ANCOVAs showed no main effect of age, body mass, and menarche, indicating that stature-adjusted bone density at the lumbar and hip site is independent of chronological age, body mass loading, and sexual maturation in our sample of young girls aged 7–15 year.

A limitation of this work is that we only tested one volume of recreational dance exposure (2 h/w), thereby preventing assessment of a dose–response effect on body composition. Another limitation is that we used a 24 h recall for assessment of caloric intake and its composition; this prevented reliable evaluation of calcium intake and, therefore, its association with bone mineral measurements. Strength of this work are the a priori analysis ensuring adequate statistical power, the estimation of impact loading in RD and RG enabling objective differentiation of the two groups in terms of osteogenic stimulus, and the use of a proper control group (physically active girls) avoiding the risk of misinterpretation of results.

In conclusion, this work showed that long-term, low-volume (2 h/w) recreational dance exposure in the form of extracurricular structured physical activity has a potential to improve the body composition of young girls in comparison with controls involved in extracurricular unstructured physical activity, when recreational gymnastics is taken as the benchmark. Findings presented herein support the exploitation of recreational dance as a cheap, easy-to-use tool to counteract the adverse effects of a sedentary lifestyle in the female pediatric population. The effect of higher volume (3–4 h/w) of recreational dance exposure on body composition in young girls is presently under investigation.

## Figures and Tables

**Table 1:** Demographic characteristics of the three groups of study participants. One-way ANOVA. Means ± SD.

Variable	Group			F_(2,62)_	*p* Value
	C (*n* = 22)	RD (*n* = 21)	RG (*n* = 22)		
Age (months)	141.5 ± 25.30	136.7 ± 26.29	126.4 ± 25.15	1.997	0.144
Body mass (kg)	42.3 ± 12.47 *	41.0 ± 11.70	33.4 ± 9.82	3.850	0.027
Stature (cm)	149.7 ± 12.91 *	147.2 ± 12.77 *	136.9 ± 11.53	6.519	0.003
BMI (kg/m^2^)	18.4 ± 3.04	18.5 ± 3.25	17.4 ± 2.21	1.051	0.356

C, physically active controls; RD, recreational dancers; RG, recreational gymnasts. BMI, body mass index. *, *p* < 0.05 vs. RG.

**Table 2:** Z-score of the three groups of study participants. One-way ANOVA. Means ± SD.

Variable	Group			F_(2,62)_	*p* Value
	C (*n* = 22)	RD (*n* = 21)	RG (*n* = 22)		
WB Z-score	−0.8591 ± 0.68637	−0.9571 ± 0.88293	−0.5905 ± 0.79051	1.218	0.303
Hip Z-score	−0.4850 ± 0.97530	−0.4944 ± 0.86872	0.0111 ± 0.83376	1.899	0.160
Spine Z-score	−0.1579 ± 0.80506	−0.3842 ± 0.96567	0.2278 ± 1.01158	2.038	0.140

C, physically active controls; RD, recreational dancers; RG, recreational gymnasts; WB, whole body.

## Data Availability

Raw data supporting reported results can be obtained from the corresponding author upon reasonable request.

## Supplementary Materials

The following supporting information can be downloaded at: https://www.mdpi.com/article/10.3390/children9030391/s1, Table S1: Descriptive statistics of DXA-measured body composition variables in the three groups of girls according to the standard DXA output. Means ± SD.
