# Supplementary material for: The Limited Impact of Low-Volume Recreational Dance on Three-Compartment Body Composition and Apparent Bone Mineral Density in Young Girls
Source: Children (Basel). 2022 Mar 10;9(3):391. doi: 10.3390/children9030391 (PMC8947180; doi:10.3390/children9030391)
Supplement: Supplementary file 1 [file children-09-00391-s001.zip › children-1610437-supplementary material.pdf]

children

MDPI

Supplementary Table S1. Descriptive statistics of DXA-measured body composition variables in the three groups of girls according to the standard DXA output. Means ± SD.

Variable	Group		
	C<br>(n=22)	RD<br>(n=21)	RG<br>(n=22)
Whole body (WB)			
Bone mineral			
WB AREA (cm^2)	1563.7 ± 243.10	1513.1 ± 255.74	1381.9 ± 215.96
WB BMC (g)	1319.4 ± 328.63	1258.7 ± 390.44	1134 ± 313.79
WB BMD (g/cm^2)	0.832 ± 0.0896	0.815 ± 0.1129	0.808 ± 0.0911
TBLH BMC (g)	936.1 ± 290.39	887.1 ± 321.55	779.1 ± 278.95
TBLH BMD (g/cm^2)	0.682 ± 0.1021	0,669 ± 0.1107	0.654 ± 0.1031
Left-arm BMC (g)	66.6 ± 24.39	67.3 ± 27.24	64.9 ± 24.57
Left-arm BMD (g/cm^2)	0.492 ± 0.0746	0.492 ± 0.0823	0.510 ± 0.0729
Right arm BMC (g)	78.2 ± 26.15	74.7 ± 29.89	69.1 ± 27.14
Right arm BMD (g/cm^2)	0.513 ± 0.0800	0.514 ± 0.0878	0.516 ±0.0822
Pelvis BMC (g)	139.6 ± 47.36	129.5 ± 51.77	117.3 ± 49.11
Pelvis BMD (g/cm^2)	0.835 ± 0.1526	0.813 ± 0.1760	0.776 ± 0.1714
Left leg (g)	230.4 ± 67.82	217.9 ± 72.65	186.7 ± 61.25
Left leg BMD (g/cm^2)	0.805 ± 0.1310	0.790 ± 0.1315	0.757 ± 0.1221
Right leg BMC (g)	227.5 ± 73.28	214.7 ± 72.34	180.6 ± 59.79
Right leg BMD (g/cm^2)	0.800 ± 0.1333	0.790 ±0.1344	0.751 ± 0.1195
Trunk BMC (g)	330.4 ± 104.80	312.5 ± 123.17	277.7 ± 108.74
Trunk BMD (g/cm^2)	0.652 ± 0.0950	0.628 ± 0.1143	0.985 ± 0.5290
FM & LSTM			
Left-arm FM (g)	635.7 ± 305.41	565.0 ± 275.46	349.0 ± 172.21
Left-arm LSTM (g)	1376.9 ± 343.20	1330.9 ± 312.15	1368.0 ± 379.25
Right arm FM (g)	635.7 ± 312.44	573.3 ± 276.47	361.1 ± 179.54
Right arm LSTM (g)	1513.1 ± 382.16	1490.8 ± 355.10	1429.1 ± 414.50
Trunk FM (g)	3989.5 ± 1934.90	3804.0 ±2007.11	2233.4 ± 1176.15
Trunk %FM	20.1±5.09	20.3±5.43	14.9±3.16
Left leg FM (g)	2658.5 ± 1071.38	2404.6 ± 978.67	1456.6 ± 647.77
Left leg LSTM (g)	4769.7 ± 1396.17	4732.2 ± 1310.82	4138.2 ± 1238.92
Right leg FM (g)	2674.7 ± 1095.26	2401.9 ± 1007.12	1468.4 ± 663.15
Right leg LSTM (g)	4790.9 ± 1473.28	4812.3 ± 1389.26	4090.9 ± 1231.70
WB FM (g)	11368.6 ± 4627.86	10498.9 ± 4460.86	6591.5 ± 2832.37
WB %FM	26.4±5.03	25.3±4.68	19.4±3.32
WB LSTM (g)	29194.2 ± 7721.31	28861.1 ± 7625.88	25447.1 ± 6872.41
TBLH FM (g)	10612.1 ± 4580.21	9749.0 ± 4427.36	5868.6 ± 2791.10
TBLH LSTM (g)	26572.1 ± 7559.03	26262.6 ± 7510.03	22936.3 ± 6711.80
Appendicular FM (g)	6622.7 ± 2766.39	5944.9 ± 2487.80	3635.1 ± 1642.00

Appendicular %FM	32.7±6.4	30.9±5.11	23.5±4.70
Special skeletal sites			
Spine			
Lumbar vertebrae (1-4) BMC (g)	36.2 ± 12.45	33.0 ± 14.81	34.7 ± 13.39
Lumbar vertebrae (1-4) BMD (g/cm^2)	0.745 ± 0.1517	0.709 ± 0.1699	0.729 ±0.1547
Hip			
Trochanter BMC (g)	4.6 ± 1.73	4.1 ± 1.89	4.1 ± 1.50
Trochanter BMD (g/cm^2)	0.670 ± 0.1096	0.591 ± 0.099	0.610 ± 0.0815
Intertrochanter BMC (g)	11.5 ± 3.54	11.5 ± 3.58	10.6 ± 2.71
Intertrochanter BMD (g/cm^2)	0.837 ± 0.1560	0.800 ± 0.1339	0.849 ± 0.1243
Femoral neck BMC (g)	2.8 ± 0.81	2.6 ± 0.85	2.6 ± 0.65
Femoral neck BMD (g/cm^2)	0.697 ± 0.1027	0.695 ± 0.1105	0.723 ± 0.0844
Ward’s area BMC (g)	0.85 ± 0.199	0.83 ± 0.161	0.88 ± 0.158
Ward’s area BMD (g/cm^2)	0.716 ± 0.1476	0.706 ± 0.1200	0.755 ± 0.1059
Hip total BMC (g)	19.0 ± 5.80	18.1 ± 6.09	17.4 ± 4.63
Hip total BMD (g/cm^2)	0.747 ± 0.1272	0,724 ± 0.1105	0.753 ± 0.0919

C, physically active controls; RD, recreational dancers; RG, recreational gymnasts; TBLH, total body less head; BMC, bone mineral content; BMD, bone mineral density; FM, fat mass; LSTM, lean soft tissue mass
